# Illegal Hunting and Bushmeat Trade around Save Valley Conservancy

**DOI:** 10.1007/s00267-025-02136-y

**Published:** 2025-03-11

**Authors:** Josephine Zisadza, Admire T. Mrewa, Amanda Khosa, Simbai A. Mutematemi, Justice Muvengwi

**Affiliations:** 1 Department of Wildlife and Safari Management, Chinhoyi University of Technology, Private Bag 7724 Chinhoyi, Zimbabwe; 2 Department of Forest Resources & Wildlife Management, Faculty of Applied Science, National University of Science and Technology, P.O. Box AC 939, Ascot, Bulawayo, Zimbabwe; 3 Department of Geography and Environmental Science, Geo-information and Earth Observation Centre, University of Zimbabwe, Harare, Zimbabwe; 4 School of Animal, Plant and Environmental Sciences, University of the Witwatersrand, Private Bag 3, Wits, 2050 Johannesburg, South Africa; 5 Save Valley Conservancy, P.O. Box 47, Birchenough Bridge, Harare, Zimbabwe

**Keywords:** Bushmeat market, Conflict, Hunters, Poaching, Retaliation, Save Valley Conservancy

## Abstract

Globally, illegal bushmeat hunting has contributed to the decline of over 300 species listed as threatened on the International Union for Conservation of Nature (IUCN) Red List and accounts for an estimated 5 million tons of wildlife harvested annually, particularly in tropical regions, placing immense pressure on biodiversity. Despite its recognized conservation threat, limited information exists on the bushmeat trade dynamics in sub-Saharan African savannas. This study conducted interviews with 133 illegal hunters and 40 anti-poaching field rangers in Southeastern Zimbabwe, using semi-structured questionnaires. We explored the characteristics, motivations, methods, species targeted, and perceptions of wildlife law enforcement in Save Valley Conservancy (SVC). Overall, illegal bushmeat hunting in SVC is mainly done by less educated and unemployed young to middle aged men (15–40 years old). The motives behind illegal bushmeat hunting mainly included household consumption (96%), the desire to raise income (96%), unemployment (78%), retaliation for wildlife induced losses (62%), culture (29%) and poor benefit sharing (8%). The common hunting methods reported were hunting with dogs (87%), and snaring (65%). Targeted animal species included impala (96%), wildebeest (53%), eland (53%), African buffalo (51%) among other 12 animal species. Illegal bushmeat hunting was generally conducted all year round. The law enforcement penalties were considered less deterrent, and most of the hunters intended to continue with illegal hunting. Measures suggested to minimize illegal bushmeat hunting in the SVC included investing and strengthening wildlife law enforcement, provision of community conservation-based incentives and enhancing environmental education and awareness.

## Introduction

Worldwide, biodiversity loss is one of the “three planetary crises” humanity is currently facing, alongside climate change and pollution (UN 2023). Among the leading drivers of this crisis is the extreme hunting of wild animals (Di Minin et al. 2021; Heurich et al. 2018; Mozer and Prost 2023; Smiley Evans et al. 2020). Globally, one in four mammal species is considered threatened with extinction (UN 2023), due to unsustainable illegal bushmeat hunting (Bennett and Robinson 2000; Fa et al. 2002; Di Minin et al. 2021). To effectively reduce the threat of hunting, it is essential to understand the underlying factors driving these high levels of exploitation with serious consequences on biological conservation (der Westhuizen and Vliet 2012; Lindsey et al. 2012).

Studies have identified diverse political, social, economic and environmental drivers of illegal bushmeat hunting, including excessive drought conditions, poor agricultural yields (Gandiwa et al. 2013), increased bushmeat demand in both rural and urban areas (Lindsey et al. 2011, 2013), and extensive road network in protected areas (Chemura et al. 2024). Other contributing factors include the need to raise educational funds, human population expansion near protected areas, and retaliation for wildlife-induced losses (Matseketsa et al. 2022; Brown and Robinson 2003; Maurice et al. 2017; Rentsch and Damon 2013a). These drivers vary across different spatial and temporal contexts, making the dynamics complex and sometimes contradictory.

To gain an in-depth understanding of the motivations of illegal bushmeat hunting, it therefore becomes important to analyze the characteristics of the offenders (Janssen et al. 2024). Indeed, characteristics like age, gender, cultural customs, and socio-economic circumstances are known to exert an influence on illegal hunters’ motivations and methods of operation (Akinsorotan et al. 2020). In most cases, illegal bushmeat hunters are considered as local, low-income, uneducated, jobless men with limited alternative income-generating opportunities (Adams et al. 2004, Lindsey et al. 2012).

The commonly used method for illegal bushmeat hunting is wire snaring (Lindsey et al. 2012). The wire to make snares is easily obtainable from fencing, burnt tyres and abandoned telephone and electricity lines (Lindsey et al. 2011a). However, illegal hunters use a wide range of methods including dogs, spotlighting (is a method where bright, focused lights are used to illuminate animals at night. The intense beam of light temporarily blinds or disorients the animals, causing them to freeze or remain still, which makes them easier to locate and target), firearms, spears, bows and arrows (Gandiwa 2011, Knapp et al. 2017, Lindsey et al. 2012). Illegal bushmeat harvesting usually occurs all year round with some seasonal variations in most protected areas (Matseketsa et al. 2022) and the levels have been found to vary across different land ownership (Duporge et al. 2018). Ungulates including impala (*Aepyceros melampus*), wildebeest (*Connochaetes taurinus*) and buffalo (*Syncerus caffer*), are among the most frequently harvested species (Rogan et al. 2017).

Illegal hunters face significant risks and costs in their activities as most bushmeat hunting in African savannas is illegal (Lindsey et al. 2012). Its elusive nature exposes the hunters to attacks by dangerous animals and injuries like punctures, wounds, and broken bones. Upon arrest the illegal hunters may be subjected to fines and other forms of punishments (Duffy et al. 2019). Regardless of having these numerous risks, illegal bushmeat hunting is prevalent in many protected areas because the potential benefits accrued by illegal hunters outweigh the costs (Knapp 2012).

To date, various bottom-up and top-down strategies have been implemented to reduce the rate and intensity of illegal hunting. While top-down measures like law enforcement are critical in reducing illegal activities and protecting wildlife resources, their effectiveness is highly debated (Duffy et al. 2019; Witter 2021). Nevertheless, it’s worth noting that, bushmeat poaching is particularly prevalent in regions characterized by inadequate law enforcement (der Westhuizen and Vliet 2012).

Generally, wildlife crimes in several countries are considered as of low priority (Lindsey et al. 2012). The gazetted penalties like warning, community service and low value fines do not reflect the value of wildlife resources being lost (Lindsey, et al. 2011b). While the judiciary systems lag in effective wildlife laws enforcement, poor field rangers’ morale often weakens the efficacy of anti-poaching efforts (Duffy et al. 2019). In most cases, they are underequipped, receive poor salaries, understaffed, and lack sufficient supervision.

In Zimbabwe, illegal bushmeat hunting is proving to be a threat to wildlife populations in numerous protected areas, and especially in the Save Valley Conservancy (hereafter SVC). Previous studies in the SVC focused on the dynamics of illegal hunting (Lindsey et al. 2011a), ecological, and economic impacts of illegal hunting in the conservancy (Lindsey, et al. 2011b). To increase our understanding of illegal bushmeat hunting in SVC, we sought to address the following questions: (1) What are the demographic characteristics of illegal hunters, motivations of long-term illegal behavior, risks and costs faced by illegal hunters, methods used for illegal hunting, commonly targeted wild animals, illegal hunting seasonality, and hunters and field rangers perceptions on the effectiveness of law enforcement in SVC?, and (2) what are the respondents suggestions on ways to reduce illegal bushmeat hunting in SVC?. Our study findings are intended to aid wildlife conservation and provide conservationists with insights on illegal bushmeat hunting in a protected area occurring in a sub-Saharan African savanna.

## Methods

### Study Area

**Fig. 1:**
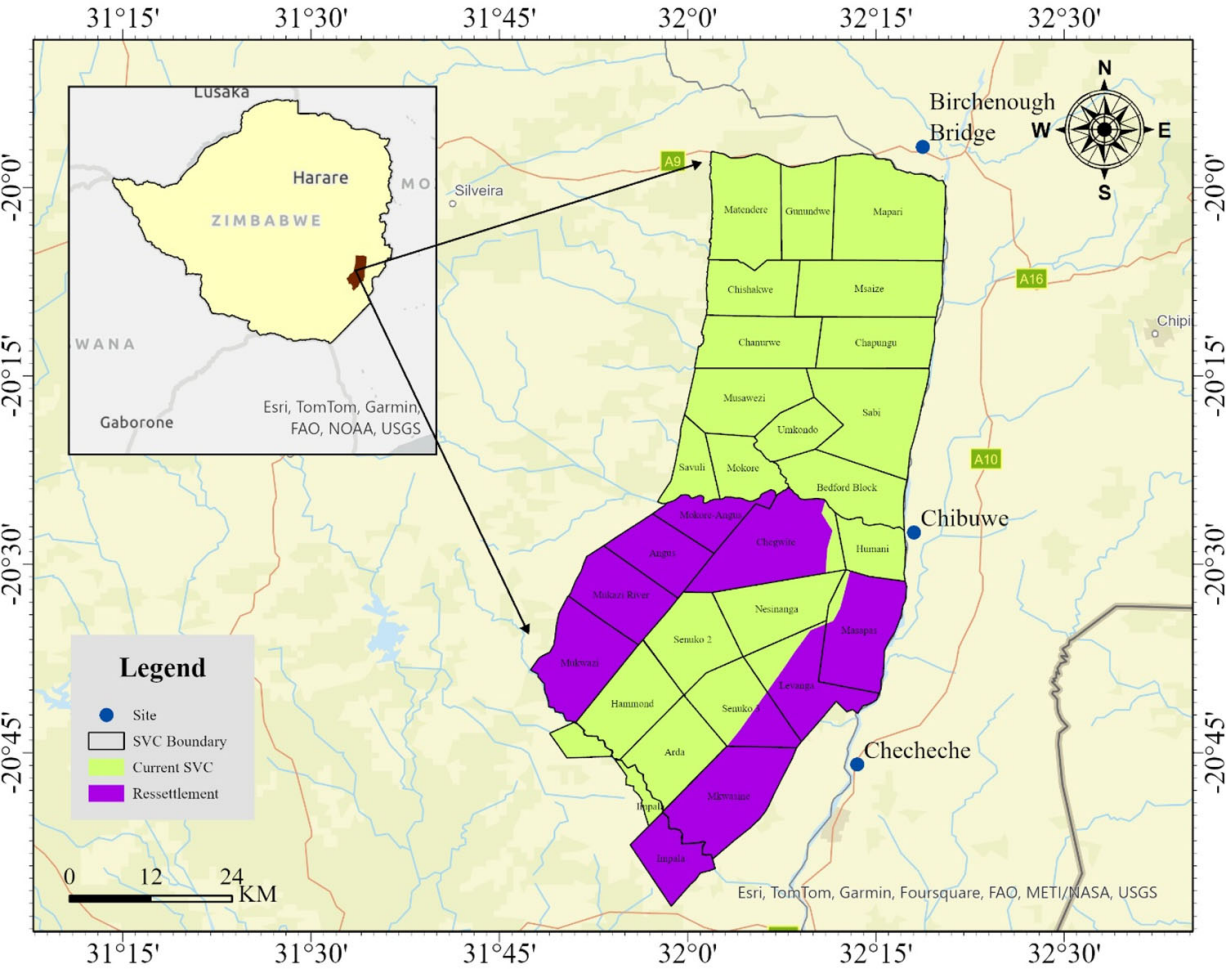
Map showing the location of the Save Valley Conservancy in Zimbabwe and the three study sites together with the resettlement areas which used to be part of the conservancy

Save Valley Conservancy is an ~3400 km^2^ cooperatively managed wildlife area, located in the South- East Lowveld of Zimbabwe (20°05S, 32°00E) (Fig. 1). Prior to April 2014, the conservancy was under cooperative private management, but it was subsequently placed under the custodianship of the Zimbabwe Parks and Wildlife Management Authority (ZPWMA) in May 2014 (Matseketsa et al. 2019). SVC was initially fenced but the fence was removed between 2000 and 2001 following “fast track” land reform programme (Lindsey et al. 2008). The conservancy lost some of its ranches including Masapas, Angus, Levanga and some parts of Senuko to subsistence farmers (Fig. 1). The resettled residents vandalized and stole the perimeter fence mainly for making snares (Lindsey et al. 2011).

Currently, SVC has seventeen functional properties, and these are home to diverse fauna and flora species. The area is characterized by a deciduous savanna woodland. The dominant woodland type is *Colophospermum mopane*, alongside other prominent woodlands like *Combretum apiculatum, Diospyros quiloensis, Kirkia acuminata, Acacia tortilis*, *Acacia erubescens*, and *Acacia nigrescens*. The local fauna boasts a diverse array of animals. Notable herbivores found in the area encompass the African buffalo (*Syncerus caffer*), waterbuck (*Kobus ellipsiprymnus*), plains zebra (*Equus quagga*), blue wildebeest (*Connochaetes taurinus*), eland (*Taurotragus oryx*), and impala (*Aepyceros melampus melampus*). The region’s wildlife also features a variety of carnivores, including spotted hyenas (*Crocuta crocuta*), leopards (*Panthera pardus*), and lions (*Panthera leo*), among other species. All the property holders are obliged to conduct anti-poaching on their properties. Trophy hunting and other natural resource related activities like fishing and photography are the income generating activities in the conservancy.

The communities that boarder the conservancy are densely populated (11–80 people/km^2^) (ZimStat, 2012). This study was carried out in villages at Chibuwe, Checheche and Birchenough. The area is located in a semiarid region five (V) in Masvingo Province which receives very little annual rainfall of 474–540 mm per annum. The area has poor-quality soils which are not suitable for agriculture. However, villagers grow small grains which are drought tolerant such as sorghum (*Sorghum bicolor*), millet (*Pennisetum glaucum*), and rapoko (*Eleusine coracana*). They also grow maize at a smaller scale. Small scale livestock production rearing domestic animals like cattle, pigs, goats and drought power animals like donkeys is also practiced. There is commercial agriculture to the south and east of SVC. Some local people get part time and/ full time jobs on these estates.

### Data Collection Methods

#### Primary data collection

##### Questionnaire surveys

Semi-structured questionnaire surveys were done with two groups of respondents, illegal hunters and field rangers. Data were collected using an interview administered questionnaire in the local language (Shona). Throughout the study, anonymity of the respondents was maintained, and it was made clear that the information collected was solely for research purposes. Respondents were informed that the surveys would be anonymous and were part of a university study assessing wildlife and rural livelihoods. The study adhered to ethical standards to ensure that the rights and welfare of the participants were protected. To gain a comprehensive understanding of the nature and extent of poaching in SVC, the questionnaire had both open-ended and closed-ended questions. Close-ended questions aimed for more precise responses, while open-ended questions allowed respondents to express their views and provide additional information on the subject.

***Illegal hunters***- Due to the cryptic nature of poaching and the targeted respondents, our study employed a multistage sampling approach to select the sampling units (poachers). The first technique used was purposive sampling to select sites near SVC with high frequency of reported poaching activity from the conservancy records. As a result, three study sites, Chibuwe, Checheche and Birchnough Bridge in three out of five districts which share boundaries with SVC emerged as major origins of many previously apprehended poachers in the Conservancy. On the second stage, simple random sampling was used to select villages at these sites. A total of thirteen villages were selected. Finally, snowball sampling was used to select respondents in the selected villages. We chose snowball sampling due to the sensitive nature of illegal bushmeat hunting, as individuals involved in these activities may be hesitant to disclose their actions openly. Snowball sampling is widely utilized in research involving hard-to-reach populations, where key individuals can refer to others who meet the study’s criteria. Recognizing that snowball sampling can introduce selection bias since participants are likely to refer others with similar characteristics or perspectives, we implemented multiple entry points for the sampling process. This involved starting with diverse key informants, such as village heads, schoolteachers, and local youth, to ensure representation across different social networks within the communities. Once introduced to our first poacher participant, we requested recommendations for additional individuals engaged in poaching activities, allowing us to broaden our sample across various network connections. In addition, captive hunters were also interviewed in the conservancy before they were transferred to police camps. A total of 133 illegal hunters were interviewed. Data collected focused on hunters’ demography, motivations of poaching, targeted species, and perceptions towards the effectiveness of SVC anti-poaching units among other aspects (Supplementary Appendix 1). In this study, ethical considerations were prioritized to protect participants involved in sensitive and potentially incriminating activities. Informed consent was obtained verbally to avoid creating any written records that could compromise participants’ identities. Before each interview, we explained the purpose of the research, the voluntary nature of participation, and the option to withdraw at any time without penalty. Anonymity was ensured by not collecting any identifying information and by using coded identifiers instead of real names in data recording and reporting. Interview locations were selected for privacy, allowing participants to speak freely without risk of exposure. These steps were reviewed and approved by the relevant ethics committee to ensure compliance with ethical standards for research involving hidden and vulnerable populations. The interview sessions ranged from 35 to 55 min in duration.

***Field rangers***- To gain a deeper understanding of the dynamics of illegal hunting in SVC, we interviewed 40 field rangers from five different properties in SVC. Data collected focused on respondents’ demography, commonly targeted species, perceptions towards the effectiveness of

SVC security measures, and possible ways to reduce illegal hunting among other aspects (Supplementary Appendix 1). The interview sessions lasted for 30–45 min.

***Field observations-*** The study involved conducting field observations, including the use of photographs as a complementary method to provide a visual appreciation of the scope and nature of bushmeat poaching. The observational data was utilized to document noteworthy features, providing insight into the realities on the ground, and assist in verifying and interpreting data collected during the survey data. Some of the photographs included livestock predation and crop raiding (serving as evidence of motivations of poaching) during the study period, hunters’ offtake, methods of transporting the bushmeat to markets etc. The method of photographs was also used in assessing the level of sabotage and extend of vandalism to SVC properties by local communities.

#### Secondary data collection

##### Court cases

We collected records of wildlife related crime court cases from Mkwasine Police Station spanning from 1 January 2015 to 31 December 2017 in order to understand how costs and penalties associated with being convicted for illegal hunting influence hunters’ decision making. We recorded the species involved and the case outcomes to understand if criminal law could be effective enough to help wildlife species conservation.

##### Data analysis

The data collected from the survey was captured and transformed into numeric codes using Excel, associating each respondent with their corresponding answers. Descriptive statistics were used in the form of Sankey plots and percentages to summarize the quantitative data from the questionnaire. The chi-squared test for goodness-of-fit was used to test whether the demographic characteristics, drivers of illegal hunting and methods used for illegal bushmeat harvesting varied across sites. A *p*-value of <0.05 was deemed statistically significant. Analysis of variance was used to examine the variance in the proportion of bushmeat allocated to different categories such as subsistence consumption, commercial use and barter trade. Logistic regression was used to determine the influence of control variables such as family size on illegal bushmeat hunting. A generalized linear mixed effects model (GLMM) was used to assess factors influencing involvement in illegal bushmeat hunting; the effects of income generated from illegal hunting, employment, state (whether arrested or free) and family size. To mitigate the potential impact of variations in sample size across study sites, frequencies were standardized, and the number of individuals represented by each percentage figure is indicated by (n). All the analyses were done using R version 4.1.3.

## Results

### Demographic Characteristics of the Illegal Hunters

**Fig. 2:**
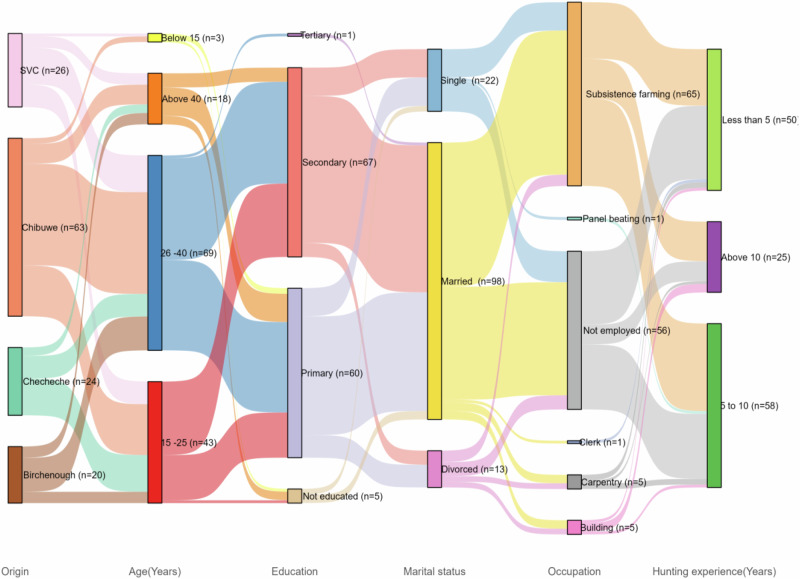
Sankey plot summarizing the age groups, level of education, marital status, profession, hunting experience of illegal hunters across sites

A total of 133 illegal hunters were interviewed. After combining data from our study sites on illegal hunters, we found that forty nine percent (*n* = 65) of the respondents originated from Chibuwe, (18%, *n* = 24) Checheche, (15%, *n* = 20) Birchenough Bridge, (18%, *n* = 24) resettled ranches in SVC. Illegal hunters’ age groups ranged from below 15 years to above 40 years (Fig. 2). The chi-squared test showed significant differences in age groups below 15 and 15–25 across regions (χ² = 8.5, df = 3, *p* = 0.037; χ² = 18.78, df = 3, *p* = 0.003, respectively; Fig. 2), whilst age groups 26–40 and above 40 were non-significant (χ^2^ = 7.51, df = 3, *p* = 0.057 and χ^2^ = 3.03, df = 3, *p* = 0.386, respectively, Fig. 2). Illegal hunters who had never attended school differed significantly across the sites (χ^2^ = 30, df = 3, *p* < 0.001, Fig. 2) whilst those who reached primary and secondary levels only were non-significant (χ^2^ = 2.98, df = 3, *p* = 0.395 and χ^2^ = 5.3483, df = 3, *p* = 0.148, Fig. 2). Approximately seven out of ten respondents (73%) were married, and the marital status of the respondents did not differ significantly across the regions; single (χ^2^ = 4.18, df = 3, *p* = 0.243, Fig. 2), married (χ^2^ = 1.47, df = 3, *p* = 0.689, Fig. 2) and divorced (χ^2^ = 1.24, df = 3, *p* = 0.744, Fig. 2). The experience of illegal hunters across regions differed significantly with less than five years (χ^2^ = 15.54, df = 3, *p* = 0.001, Fig. 2), 5 to 10 years (χ^2^ = 9.75, df = 3, *p* = 0.021, Fig. 2) and above 10 years (χ^2^ = 39.27, df = 3, *p* < 0.001, Fig. 2). The number of illegal hunters practicing different occupations did not differ significantly across the sites for subsistence farming (χ^2^ = 0.77, df = 3, *p* = 0.856, Fig. 2), building (χ^2^ = 5.36, df = 3, *p* = 0.147, Fig. 2) and carpentry (χ^2^ = 6, df = 3, *p* = 0.112, Fig. 2). Only panel beating differed significantly across the regions. Illegal hunters across the sites looked after many household members 5.85 + 0.22 (Chibuwe), 6.04 + 0.30 (Checheche), 7.9 + 0.31 (Birchenough Bridge) and 6.15 + 0.41 (SVC resettlements). The illegal hunters’ number of household members did not differ significantly across the sites (χ^2^ = 0.42, df = 3, *p* = 0.936, Fig. 2).

### Motivations for Illegal Hunting

Ninety six percent (*n* = 128) of the interviewed illegal hunters engaged in illegal hunting to obtain meat for household consumption to meet protein needs. Hunting for household need did not significantly differ across the sites (χ^2^ = 0.48, df = 3, *p* = 0.923). In addition, the majority of the respondents (93%, *n* = 124), acknowledged selling bushmeat to generate income. Engaging in bushmeat trade did not differ significantly across the sites (χ^2^ = 2.26, df = 3, *p* = 0.520). Retaliation for wildlife induced loses (including crop raiding and livestock predation) (62%, *n* = 83), unemployment (78%, *n* = 104) were also frequently cited as motivators for illegal hunting.

The driving force of retaliation differed significantly across sites (χ^2^ = 10.75, df = 3, *p*-value = 0.013), while unemployment was non-significant (χ^2^ = 1.97, df = 3, *p*-value = 0.580). Poor benefit sharing and culture were also cited as motivators of illegal hunting and these differed significantly across study sites (χ^2^ = 47.14, df = 3, *p* < 0.001) and (χ^2^ = 27.68, df = 3, *p* < 0.001) respectively.

### Bushmeat Trade

**Fig. 3:**
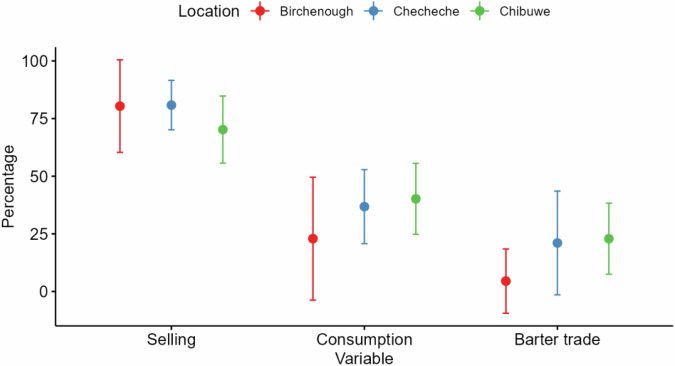
The use of harvested bushmeat by illegal hunters as a proportion across sites. Dots are means, and error bars represent 95% confidence intervals

Most illegal bushmeat hunting across sites was aimed towards the sale of bushmeat rather than subsistence provision and barter trade (Fig. 3). High proportion to bushmeat sales can be attributed to high monthly income the hunters earn.

Some Illegal hunters used illegal hunting as the main source of income and it had higher returns than selling agriculture produces (20%), livestock production (25%) and venturing into small business (14%). Most of the respondents received bushmeat orders from their customers (61%, *n* = 81). An average of 2.49 ± SE 0.36 (Chibuwe), 1.66 ± SE 0.38 (Checheche), 3.65 ± SE 0.31 (Birchenough Bridge) and 3.76 ± SE 0.89 (SVC resettlements) buyers were placing orders per hunting execution. The number of people who placed orders across sites did not differ significantly (χ^2^ = 1.04, df = 3, *p* = 0.791). The price of the bushmeat did not vary across sites; with a portion of bushmeat weighing approximately 1 kg going for US$ 2.00. The income gained from sales is used to buy groceries (94%, *n* = 125), pay hospital bills (41%, *n* = 54), pay school fees (72%, *n* = 95) and buy building material (21%, *n* = 28). For illegal hunters who did barter trade they were receiving goods like maize, sorghum and millet in return. A small percentage eventually traded barter items for money (11%, *n* = 14), however most illegal hunters consumed barter items.

The income gained from selling the bushmeat, employment status and the state of the illegal hunter were significant in explaining illegal hunters’ intention to hunt in SVC again across all sites (Table 1). Illegal hunters who received high income from bushmeat sales had the intention to hunt in SVC again (Table 1). In addition, both part time and unemployed illegal hunters had the intention to hunt in SVC again (Table 1). Free illegal hunters also showed high intention to hunt in SVC again (Table 1).

**Table 1:** Generalized linear mixed-effects model of predictors of individuals’ intention to hunt in SVC again (with intention as a binary response variable)

Parameters	Estimate Std.	Std. Error	z value	Pr(>|z|)
(Intercept)	0.5184	1.274	0.407	*p* = 0.684
State (Free)	3.6225	0.8985	4.032	*p* < 0.001
Employment status (Employed)	−2.7735	1.2142	−2.284	*p* = 0.022
Income (High)	2.4462	0.8423	2.904	*p* = 0.004
Family size	−0.3006	0.2111	−1.424	*p* = 0.154

Location (four sites: Chibuwe, Checheche, Birchenough Bridge, and SVC resettlements) is included as a random effect. Interactions between the variables were not significant

### Methods, Target Animals and Seasonality

**Fig. 4:**
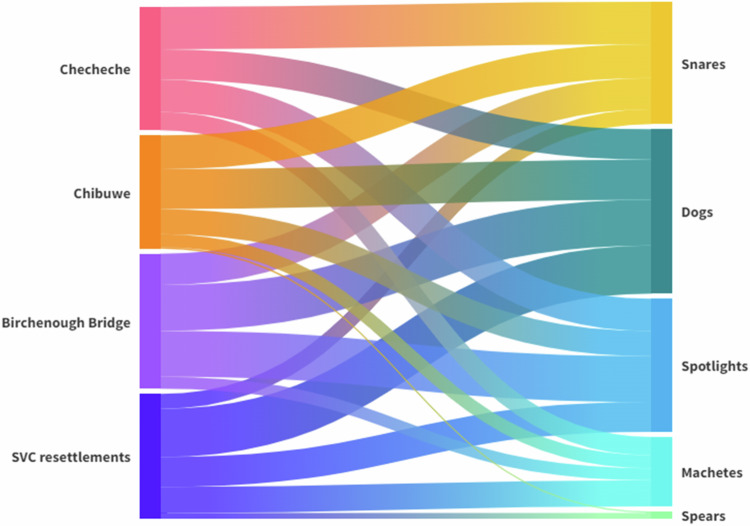
Methods used by illegal hunters across sites when hunting in Save Valley Conservancy, Zimbabwe

Illegal hunters used a variety of methods in harvesting the bushmeat, dogs (86%, 114), snares (65%, *n* = 87), spotlighting (78%, *n* = 104), machetes (56%, *n* = 74), spears (5%, *n* = 7), bows and arrows. In some cases, snares and dogs were used concurrently (i.e. set snares and use dogs to drive animals into snares). The frequency of use of different hunting methods differed significantly across sites, dogs: χ^2^ = 9.53, df = 3, *p* = 0.023, snares: χ^2^ = 26.79, df = 3, *p* < 0.001, spotlights: χ^2^ = 14.75, df = 3, *p* = 0.002, machetes: χ^2^ = 14.72, df = 3, *p* = 0.002 and spears: χ^2^ = 25.8, df = 3, *p* < 0.001 (Fig. 4). The highest use of dogs for example was from SVC resettlements (100%, *n* = 26), and the least in Checheche (63%, *n* = 15). Across the study sites, illegal hunters would hunt in small teams of 3.65 ± SE 0.16 (Chibuwe), 3.41 ± SE 0.21 (Checheche), 4.25 ± SE 0.20 (Birchenough) and 2.92 ± SE 0.19 (SVC resettlements). The illegal hunters would hunt with many dogs per hunting trip: 3.92 ± SE 0.28 (Chibuwe), 3.25 ± SE 0.51 (Checheche), 6.65 ± SE 0.59 (Birchenough Bridge) and 4.65 ± SE 0.43 (SVC resettlements).

The wire to make snares was obtained from the conservancy perimeter fence (38%), old telephone lines (10%), people’s gardens (10%) and sometimes traded with bushmeat (4%). Spotlights were used to blind animals in the dark and were cheap in local shops, costing US$ 3.00 on average. Illegal hunters selected hunting areas in SVC based on general wildlife abundance (88%, *n* = 117), abundance of preferred species (31%, *n* = 41) and proximity to home (57%, *n* = 76). The use of dogs and spotlights were the most dominant methods across sites (Fig. 4) and the illegal hunters using dogs selected hunting areas in SVC based on proximity to home (62%), wildlife abundance (86%) and abundance of preferred species (30%). All the methods used for illegal hunting were inexpensive, locally available (41%, *n* = 54) and they were effective and maximize returns (59%, *n* = 79).

Field rangers (100%, *n* = 40) acknowledged that most animals, especially herbivores were at risk from poaching. This was also supported by data from many illegal hunters who highlighted that they targeted most animal species despite them being small-, medium- or large-sized (Table 2).

**Table 2:** Animal species commonly targeted by illegal hunters in Save Valley Conservancy (SVC), Zimbabwe

Animal species	Scientific name	Number of respondents	Percentage
Impala	*Aepyceros melampus*	128	96
Bushpig	*Potamochoerus larvatus*	98	74
Warthog	*Phacochoerus africanus*	95	71
Blue wildebeest	*Connochaetes taurinus*	71	53
Eland	*Taurotragus oryx*	71	53
African buffalo	*Syncerus caffer*	68	51
Burchell’s zebra	*Equus quagga*	67	50
Bushbuck	*Tragelaphus scriptus*	62	47
Waterbuck	*Kobus ellipsiprymnus*	61	46
Grysbok	*Raphicerus melanotis*	60	45
Sable	*Hippotragus niger*	59	44
Giraffe	*Giraffa camelopardalis*	59	44
Duiker	*Sylvicapra grimmia*	74	56

Total percentage exceeds 100 because the illegal hunters were allowed to give multiple answers

**Fig. 5:**
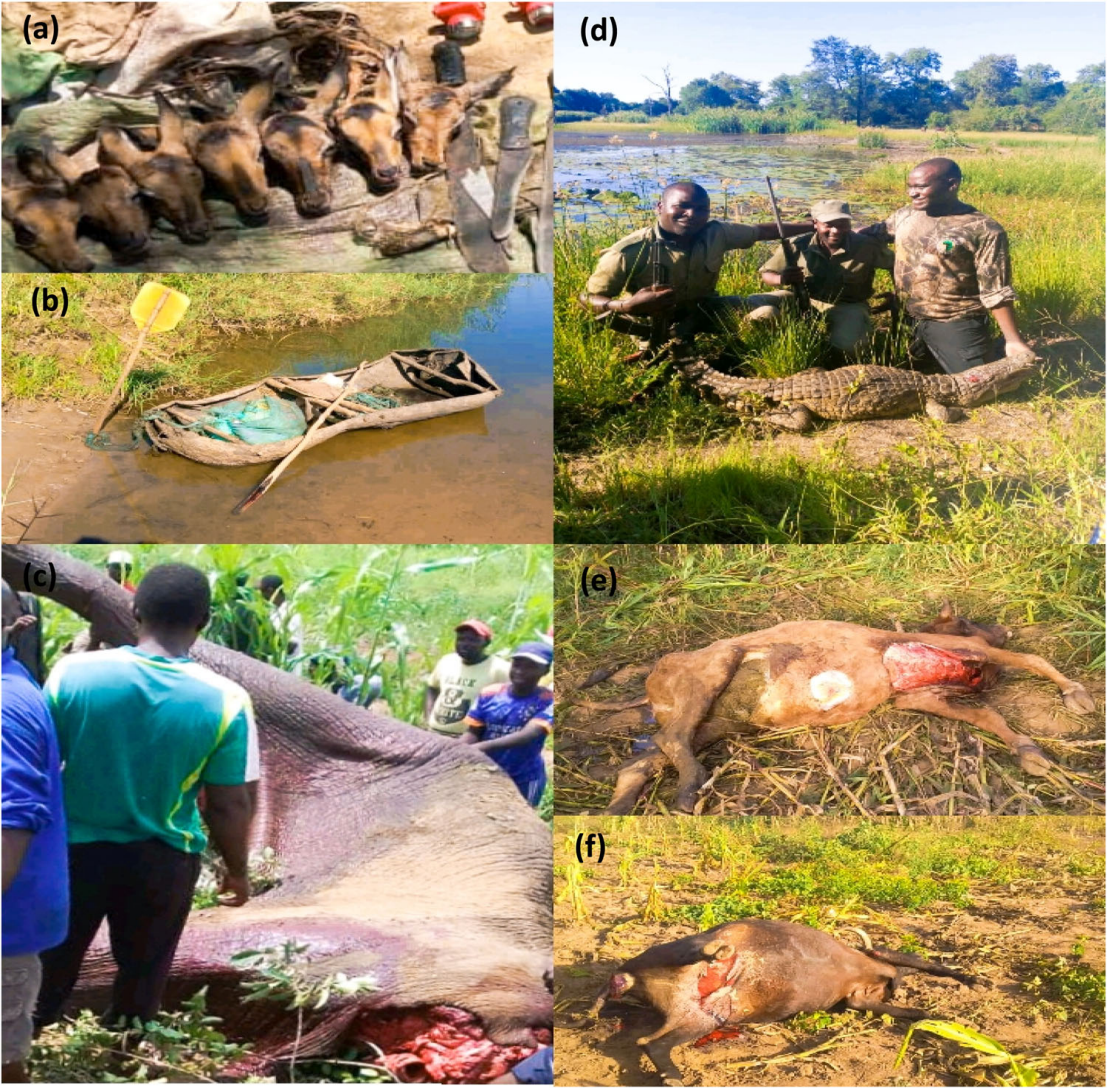
**a** Offtake by three illegal hunters, **b** canoe used by illegal hunters to transport bushmeat cross Save River, **c** elephant killed for crop raiding in adjacent communities, **d** crocodile killed for livestock and human attacks along Save River, **e**, **f** cattle killed by lions from SVC in adjacent communities during the study period

Illegal hunters usually killed many animals per hunting trip 4.01 ± SE 0.15 (Chibuwe), 4.45 ± SE 0.20 (Checheche), 5.35 ± SE 0.33 (Birchenough Bridge) and 3.34 ± SE 0.19 (SVC resettlements). With such unsustainable offtakes, majority of illegal hunters perceived that animal population densities were decreasing in SVC (71%, *n* = 95). A small percentage considered the populations as fluctuating (29%, *n* = 39). These views were attributed to more hunting effort for illegal hunters (53%, *n* = 71) and more hunting pressure from numerous illegal hunters (20%, *n* = 26). The market for bushmeat included villages (93%, *n* = 124) and local restaurants and butcheries (62%, *n* = 82). A small percentage (37%) incurred transport cost, when transporting the bushmeat from the conservancy to their respective markets. In many cases illegal hunters would use carriers to ferry the harvested meat or carcasses. During the rainy season, illegal hunters from Chibuwe, Checheche and SVC resettlements crossed the Save river using canoes (Fig. 5b).

Majority of illegal hunters (89%, *n* = 119) harvested game all year round, while a very small percentage (8%, *n* = 10) and (3%, *n* = 4) hunted seasonally, i.e. wet and dry season only, respectively. High bushmeat demand (62%, *n* = 83), poverty and unemployment (74%, *n* = 98) were the frequently cited reasons for perennial illegal hunting.

### Anti-poaching Effectiveness

All the properties in Save Valley Conservancy maintain ranger staff, although their organization varied depending on the property. However, despite the variations, interviews with the field rangers revealed that there is heavy poaching pressure, and they are not capacitated enough for the job. Majority of the illegal hunters perceived that the security in their respective properties was poor to effectively deter illegal bushmeat hunting. They field rangers are understaffed (48%, *n* = 19), lack sufficient equipment and resources (53%, *n* = 21) to adequately patrol, respond to and investigate illegal hunting cases. On some properties there were 6 or less field rangers to patrol areas larger than 11,000 hectares.

Majority of the illegal hunters (61%, *n* = 81) perceived most SVC anti-poaching units (field rangers) as not effective. This was attributed to several reasons which included few field rangers who are underequipped, and some rangers are involved in poaching syndicates. The co-operating field ranger would give information on where to hunt (29%), allow illegal hunters to hunt after receiving a bribe (11%) and would partner in hunting and selling (13%). Only a few (38%, *n* = 51) viewed the anti-poaching units as effective.

### Wildlife Law Enforcement

There was evidence of repeated arrest as 54% (*n* = 72), illegal hunters that were arrested twice or even more for poaching. Majority of the hunters reported that the punishments they are given after being arrested were relatively easy (50%, *n* = 66). These included warning and caution (28%, *n* = 37), community services and fines (19%, *n* = 25) and short prison sentences (26%, *n* = 34). Field rangers (100%, *n* = 40) highlighted that judiciary penalties were not deterrent enough to curb illegal hunting

Additionally, we found 260 cases from Chiredzi magistrate court involving 351 defendants. 38% of the defendants were convicted for setting up wire snares (in line with the Copper Act 1962: which prohibits dealing in copper without a license) and not necessarily for the illegal hunting. 42% of the cases involved species commonly hunted for bushmeat which include the African buffalo (*Syncerus caffer*), duiker (*Sylvicapra grimmia*), eland (*Taurotragus oryx*), warthog (*Phacochoerus africanus*), zebra (*Equus quagga)*, bushpig (*Potamochoerus larvatus*), wildebeest (*Connochaetes taurinus*) and impala (*Aepyceros melampus*) among many ungulates. Not all illegal hunters were given penalties. In 20% of the cases, the accused (illegal hunter) could not be located (i.e. does not show up on given court dates). 10% of the cases were withdrawn before plea due to lack of enough evidence. In 5% of the cases, the offenders were warned, cautioned and discharged because either they were juveniles or first-time offenders. 40% of the offenders were given community service and fines in lieu of prison sentences (which they serve if they cannot perform the community service or pay the stipulated fine). The fines ranged from US$20-200 per offence and the community service had a maximum of 375 h. Prison sentences ranged from five days to 8 months.

### Strategies for Reducing Illegal Bushmeat Hunting in the Save Valley Conservancy

Majority of the illegal hunters highlighted that implementation of viable conservation based projects and incentives (80%) which can help in employment creation and provide direct benefits to the local communities can play an essential role in reducing illegal bushmeat hunting. In addition, other strategies suggested by the illegal hunters include erection of electric fence (83%), improving wildlife law enforcement in SVC through improving anti-poaching (39%) and providing meat handouts (16%) to adjacent communities.

## Discussion

### Demographic Characteristics

More than ten years on, the present study still suggests that illegal bushmeat hunting is common and widespread in the SVC, corroborating previous findings (Lindsey et al. 2011a, 2011b). Most illegal hunters in SVC mainly constitute of young to middle aged men between 15 to 40 years old, which is similar to a study that was carried out in the same landscape more than 10 years ago (Lindsey et al. 2011a). Only a few individuals younger than 15 years old or over 40 years old engage in illegal bushmeat hunting. Most illegal hunters are less educated, unemployed and depend heavily on subsistence farming, a finding that is similar to other studies (Rogan et al. 2018; Lindsey et al. 2013). Furthermore, following the country’s persistent economic crisis and unfavorable agricultural conditions in the semi-arid lowveld, our study shows that 62% of the illegal hunters have been practicing illegal bushmeat hunting in the conservancy for relatively long periods to sustain their livelihoods.

### Drivers of Illegal Bushmeat Hunting

This study revealed that food insecurity drives illegal bushmeat hunting in SVC, with 96% of hunters citing household protein needs as their primary motivation. Globally, food insecurity and rampant wildlife exploitation are highly interlinked (King 2014; Nasi et al. 2011; Rentsch and Damon 2013b; van Velden et al. 2018; Wilkie et al. 2016). To address food insecurity in surrounding communities, SVC authorities implemented a game cropping and meat distribution scheme in 2009, though the initiative proved short-lived (Lindsey et al. 2011). The game cropping scheme’s limited focus on elephant meat failed to address both the quantity and diversity of local protein demands, resulting in continued illegal hunting (Le Bel et al. 2013).

Our study also highlighted that illegal bushmeat hunting in SVC is driven by retaliation to compensate for wildlife induced losses. Farmers often resort to illegal hunting as a retaliatory response to wildlife damage, particularly to protect livestock from predation (Corlett 2007; Hope 2002; Zahler et al. 2004) and crops from raiding animals (Bashari et al. 2018, Matseketsa et al. 2019). In SVC, the resettlement and fence removal during the 2001 land reform heightened human-wildlife conflict, with instances of crop raiding by animals like elephants, buffalos, elands, and livestock predation by crocodiles and lions becoming more frequent (Fig. 5c–f). Recent findings indicate a surge in the elephant population within SVC (Khosa et al. 2023), leading to a rise in crop raiding incidents and casualties (e.g., nine fatalities recorded in 2022). Inadequate compensation by responsible property owners for the affected communities could have contributed to the escalation of illegal hunting as was observed in other studies (Gayo et al. 2021; Inskip et al. 2014).

Moreso, our study highlighted that poor benefit sharing also drive illegal bushmeat hunting in the SVC. Many local communities were displaced to pave the way for the creation of SVC when the main land use was still cattle production (Lindsey et al. 2008). However, the local communities haven’t been getting equitable benefits from SVC conservation initiatives, but rather only bear the brunt of living near the protected area which was also highlighted in a previous study in the same landscape (Matseketsa et al. 2019). A study by Hübschle (2017) ascertained that displacement and dispossession is a significant element influencing involvement in illegal bushmeat hunting. Also, Harrison et al. (2015) found that resistance towards protected areas functioned as a crucial motivation for illegal hunting. Other studies argued that preventing local communities from harvesting wildlife without offering alternatives (Eliason 2003; Von Essen et al. 2014; Yamagiwa 2003) or ensuring equitable revenue distribution (Harrison et al. 2015; van Velden et al. 2020) is unjust and it fuels illegal hunting.

### Illegal Bushmeat Trade Dynamics

Bushmeat was sold at relatively low prices, US$ 2/kg which is way below the average price of alternatives such as beef in the local market, US$ 5/kg. The present study reveals that the local communities surrounding SVC find bushmeat as a cheap lucrative protein alternative, a finding similar to Lindsey et al. (2011a), and occasionally place bushmeat orders to known hunters. Placement of bushmeat orders in turn boosts the hunters’ efforts and amplifies harvesting pressure as the hunters would be working on targets. The barter trade of bushmeat that we observed in this study is not unique (Lindsey et al. 2011a, 2013). The barter trade mostly involved three common cereal crops, sorghum (*Sorghum bicolor*), millet (*Pennisetum glaucum*), and rapoko (*Eleusine coracana*) which they either sell to generate income or consume at household level.

Most of the illegal hunters highlighted that the proceeds they get from illegal bushmeat sales exceeds the income they would generate from other alternative income sources such as agricultural sales, small business, and livestock sales. This is similar to other studies where illegal hunters got more financial rewards from hunting than other sources of income (Hancock et al. 2017; Mancini et al. 2011; Knapp 2012). Our findings revealed that even those with part-time and full-time employment were involved in illegal hunting to increase their income. The monthly income gained by most of the illegal hunters ranged from US$ 50 to above US$ 200 which is way lower than the US$ 400 gained by illegal hunters in western Serengeti (Knapp 2012) and comparable to US$ 300 gained by professionals like teachers, nurses, and police officers in Zimbabwe.

### Illegal Hunting Methods, Target Animals and Hunting Seasonality

Although illegal hunters predominantly used wire snares in the SVC landscape (Lindsey et al. 2011a), our current study indicates that spotlighting at night has become the more common method. While wire snares are relatively easy and inexpensive to obtain (Lindsey et al. 2011), hunters seem to be moving away from their use particularly those made from telephone lines due to the Copper Act (Copper Control Act, 2016), which imposes severe penalties upon detection and arrest. While snares can be managed and removed through methods like snare sweeps, the increasing use of spotlights presents a greater challenge for management and anti-poaching teams, making it more difficult to monitor and curb illegal hunting effectively in the SVC landscape.

A diverse range of animal species, varying in body size, were identified as the primary targets of illegal hunters, including impala, wildebeest, zebra, and buffalo. These findings align with previous studies, which also reported similar species as being the most frequently hunted (Kümpel et al. 2009; Gandiwa, 2011; Gill et al. 2012; Rogan et al. 2017). Differences in targeted species preferences across study sites are influenced by variations in species availability, consumer taste, and demand for specific animals (Lindsey et al. 2011; Lindsey et al. 2013). Additionally, the relative abundance of wildlife in the Save Valley Conservancy (SVC) likely plays a significant role, as herbivore species with higher population densities were more frequently cited as preferred targets.

A previous study in the SVC considered illegal bushmeat hunting as a seasonal activity (Lindsey et al. 2011a). However, our current results suggest that illegal hunters in the SVC hunt all year round. Similarly, Nuno et al. (2013) observed that bushmeat hunting was aseasonal in the Serengeti, Tanzania. Aseasonal illegal hunting may lead to extreme negative effects as wildlife populations in SVC are increasingly decreasing. Interestingly, majority of the illegal hunters showed high awareness of the shrinking animal populations, but however continue to unsustainably exploit the species. Elsewhere, other authors also reported that local communities would continue to knowingly exploit even endangered species when their other needs are immediate (Alvard 1993).

### Anti-poaching Effectiveness

The present study suggests that the anti-poaching teams in SVC are not effective in reducing the rate and intensity of illegal hunting. The primary reasons for this ineffectiveness highlighted by our study include understaffing, poorly equipped and less morale for the job. Indeed, a previous study in the same landscape highlighted similar reasons, for example poor investment in antipoaching (Lindsey et al. 2011b), with some of the field rangers engaging in illegal hunting, exacerbating the hunting pressure. On the African continent, the anti-poaching approach has been largely criticized as “green militarization” of conservation which is less effective in the long run (Duffy et al. 2019), however, this is highly debatable. In much bigger properties like SVC, the respective property owners often face budgetary constraints, making it extremely difficult and costly for them to effectively manage and patrol their ranches.

### Wildlife Law Enforcement

The results of our study suggest that, despite the punitive nature of wildlife laws such as the 2011 legislative amendment to Zimbabwe’s Parks and Wildlife Act (Chapter 20:14, Section 128.1; Veritas 2018) their enforcement remains questionable. The court records we collected highlighted that the probability of illegal hunters being charged a heavy penalty is very low. Other studies have also observed a lack of deterring penalties for bushmeat poaching (Nellemann et al. 2014; Mozer and Prost 2023; Duffy et al. 2016). Our study highlighted that majority of illegal hunters had records of recidivism (re-offending) and yet intended to continue with illegal bushmeat hunting. Given the hyperinflation in the country, fines for illegal hunting can be way too low. Other studies in the southeastern lowveld (Gandiwa et al. 2013, 2014) and African savannas (Rogan et al. 2018), highlighted that the punishments and penalties given to illegal hunters, particularly in Southern Africa are less deterrent (Matseketsa et al. 2022), and do not reflect the value of the resources being lost.

### Limitations of the Study

Since our study involved illegal hunters, this could have limited the fullness of information obtained. Respondents may deliberately withhold information or provide misleading answers due to fear of legal consequences, social stigma, or potential repercussions from authorities (Solomon et al. 2007). This ‘response bias’ can lead to underreporting illegal activities and potentially skewed research findings (Gavin et al. 2010). The reliability of interview responses concerning illegal activities, particularly bushmeat hunting, presents significant methodological challenges (Nuno and St. John 2014).

## Conclusion

Illegal bushmeat hunting in SVC is largely driven by socio-economic factors like food insecurity and unemployment. To address these issues and promote sustainable wildlife management, we recommend three key strategies: improving anti-poaching measures, implementing conservation incentives, and enhancing community education. Strengthening law enforcement can be achieved by centralizing the anti-poaching unit, recruiting and training additional rangers, increasing patrol efforts, and improving ranger compensation. Additionally, community-based conservation incentives could provide economic benefits to local households, directly linking their livelihoods with wildlife conservation and reducing the financial pressures that drive poaching. Finally, SVC should implement widespread conservation education campaigns to foster awareness and positive community engagement with conservation goals. Together, these strategies can help alleviate poverty-driven poaching pressures and strengthen community support for wildlife protection in SVC.

## Supplementary information

Supporting information

## Data Availability

The data belongs to Save Valley Conservancy and it can only be requested through the organization.
