# Supplementary material for: Illegal Hunting and Bushmeat Trade around Save Valley Conservancy
Source: Environ Manage. 2025 Mar 11;75(10):2722–35. doi: 10.1007/s00267-025-02136-y (PMC12457532; doi:10.1007/s00267-025-02136-y)
Supplement: Supplementary file 1 — Supporting information [file 267_2025_2136_MOESM1_ESM.docx]

APPENDIX 1

**QUESTIONNAIRE FOR POACHING IN SAVE VALLEY CONSERVANCY**

**SECTION A: DEMOGRAPHIC DATA**

Please tick in the appropriate box against each item

1. Age

Below 15 15 to 25 26 to 40 Above 40

2. Gender

Male Female

3. Marital status

Single Married Engaged Divorced

4. Highest educational level

Primary Secondary Tertiary N/A

5. For how long have you been poaching?

Less than 5years 5- 10 years above 10 years

6. Number of people in your household?

7. What do you do for a living? _____________________________________________________________________________________________________

8. Where do you stay? _____________________________________________________________________________________________________

9. Who is the chairman? ______________________________________________________________________________________________________

**SECTION B**

10. What are your reasons for poaching in SVC?

Food Retaliation Culture Poverty Bush-meat trade Poor benefit sharing Unemployment

Other: ___________________________________________________________________________________________________

11. How many will you be when hunting?

12. What are your primary methods of poaching?

Snares Dogs Pit Traps Spotlight Bow &arrow

Other: ______________________________________________________________________________________________________

13. Number of snares and dogs. ______________________________________________________________________________________________________

14. Why do you prefer to use these methods?

______________________________________________________________________________________________________

15. What are your target species? ______________________________________________________________________________________________________

16. Do you sometimes hunt as per orders? Yes No

17. Approx., how many people place an order?

18. Species with high demand when buyers are placing their orders__________________________________________________­­­­­­­­­­­­­­­­_______________________________________________

19. Number of animals per hunting trip?

20. On average, how many days do you spent hunting per week?

21. Can you list months you hunted in the lasts 12 months? ______________________________________________________________________________________________________

22. What are your reasons for hunting during these months? ______________________________________________________________________________________________________

23. How do you select hunting area?

Proximity to home Wildlife abundance Abundance of preferred species

Other. ___________________________________________________________________________________________________

24. In your own opinion is animal population increasing/decreasing?

Increasing decreasing

25. Reasons for your answer above? ______________________________________________________________________________________________________

26. According to your experience, is poaching a difficult activity?

Yes
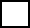
 No

27. Reasons for your answer above?

____________________________________________________________________________________________________________________________________________________________________________________________________________

28. How many times have you been arrested because of poaching?

Never
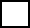
 Once
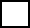
 Twice
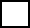
 Thrice
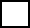
 More than thrice
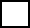


29. What do you do with the bushmeat you obtain?

Sell
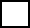
 Eat
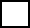
 Barter
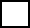


Other: ­­­­­­­­­­­­­­­­­______________________________________________________________________________________________________

30. What percentage is primarily hunted to sell the meat, subsistence use (eat) and barter?

Sell­­­______ Subsistence use________ Barter__________

30. If “Barter”, what will you be receiving in return?

____________________________________________________________________________________________________________________________________________________________________________________________________________

31. How much do you earn per month through selling barter items?

____________________________________________________________________________________________________________________________________________________________________________________________________________

32. How long does it take to sell after a hunting trip?

Less than 1hour
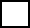
 1-10hrs
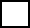
 11-24hrs
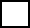
 more than 24hrs
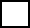


33. Do you incur any transport costs when selling the meat? Yes
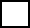
 No
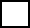


34. Mode of transport? ______________________________________________________________________________________________________

35. Location of the game meat market? ______________________________________________________________________________________________________

36. What are risks and costs of holding bushmeat stocks? ______________________________________________________________________________________________________

37. Approximately how much do you sell the meat per kg? ______________________________________________________________________________________________________

38. Basically, how much do you earn per month through illegal hunting? _____________________________________________________________________________________________________

39. How do you spend the income you get from selling bushmeat? ______________________________________________________________________________________________________

40. How much do you earn per month through the following activities?

Agricultural sales
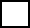
 Livestock sales
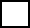
 Small business
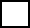
 N/A
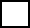


41. What are the most common punishments you are usually given after you get caught?

_____________________________________________________________________________________________________

42. In your own opinion, are these punishments harsh? Yes
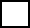
 No
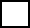


43. Reasons for your answer above? _____________________________________________________________________________________________________

_____________________________________________________________________________________________________

44. Does the money you get from bushmeat sale, surpass fines? Yes
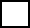
 No
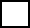


45. Upon failure to pay the fine, who will pay it for you? Relatives
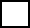
 Friends
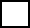
 Other

46. Where do they get the money from? Illegal hunting
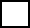
 Other sources
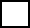


47. Where do you usually hunt? _____________________________________________________________________________________________________

48. According to your own point of view, do you think SVC anti-poaching units’ patrols are that effective?

Yes
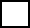
 No
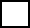


49. On a scale of 1-5, how strong is the effectiveness you mentioned above? 1
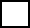
 2
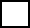
 3
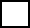
 4
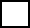
 5
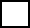


50. Reasons for anti-poaching effectiveness view ____________________________________________________________________________________________________

51. From your experience, between the ranger and wild animals, which one do you fear the most?

Ranger
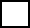
 Wild animals
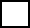


52. Can you lists the animals you fear when poaching? ­­­­­-_____________________________________________________________________________________________________

53. Do you receive any form of hunting assistance from the scouts? Yes
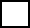
 No
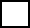


54. If “yes”. What form of assistance do you receive? ____________________________________________________________________________________________________________________________________________________________________________________________________________55. Do you have any intention to hunt in SVC again? Yes
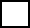
 No
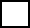


56. From your point of view, how best can Save Valley Conservancy address poaching? ________________________________________________________________________________________________________________________________________________________________________________________________________________________________________________________________________________________________________________________________________________________________________________________________________________________

**NOTES**.

_________________________________________________________________________________________________________________________________________________________________________________________________________________________________________________________________________________________________________________________________________________________________________________________________________________________________________________________________________________________________________________________________________________

APPENDIX 2

**QUESTIONNAIRE FOR POACHING IN SAVE VALLEY CONSERVANCY**

**SECTION A: DEMOGRAPHIC DATA**

Please tick in the appropriate box against each item

1. Age ____________

2. Marital status

Single Married Engaged Divorced

3. Number of people in your household?

4. Where do you stay (village of origin)? ____________________

5. Who is the chairman? _________________________

6. How long have you been in the anti- poaching sector? __________years

7. Do you personally know some of the bushmeat hunters?

Yes No

8. What is your designated employment position?

Field ranger Senior ranger Area manager

9. Which property/ranch do you work for? ________________

**SECTION B**

10. In your own view, is bushmeat poaching a serious threat to wildlife populations? Yes No

11. What do you think are the motives behind bushmeat poaching in SVC?

Food Retaliation Culture Poverty Bush-meat trade Poor benefit sharing Unemployment

Other: ___________________________________________________________________________________________________

12. The size of poaching groups when hunting?

13. What are the primary methods of hunting poachers mostly use?

Snares Dogs Pit Traps Spotlight Bow &arrow

Other: ______________________________________________________________________________________________________

14. Number of snares and dogs. ______________________________________________________________________________________________________

15. In your own view, why do they prefer to use these methods?

______________________________________________________________________________________________________

16. What are the most targeted species? ______________________________________________________________________________________________________

17. Number of animals per hunting trip?

18. How often do you think an individual comes to poach in a month? _____________________________

19. Can you list months which hunters frequently poach? ______________________________________________________________________________________________________

20. What do you think are the reasons for hunting during these months?_______________________________________________________________________________________________

21. How hunters select hunting area?

Proximity to home Wildlife abundance Abundance of preferred species

Other. ___________________________________________________________________________________________________

22. In your own view, are animal populations increasing/ decreasing?

Increasing decreasing

23. Reasons for your answer above? ____________________________________________________________________________________________________________________________________________________________________________________________________________

24. Have you arrested the same poacher twice or more because of poaching?

Yes
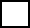
 No
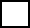


25. What do you think are the reasons promoting these repeated arrests? ____________________________________________________________________________________________________________________________________________________________________________________________________________

26. What are the most common punishments usually given to poachers after they’re caught?

_____________________________________________________________________________________________________

27. In your own opinion, are these punishments deterrent enough to curb poaching? Yes
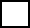
 No
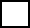


28. Reasons for your answer above? ____________________________________________________________________________________________________________________________________________________________________________________________________________

29. Does the money hunters get from bushmeat sales, surpass fines? Yes
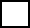
 No
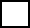


30. Upon failure to pay the fine, who will pay it for them? Relatives
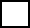
 Friends
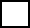
 Other

31. Where do they get the money from? Illegal hunting
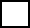
 Other sources
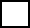


32. Is the size and structure of ranger units at your station good enough to deter poaching?

Yes No

33. Reasons for your answer above? ____________________________________________________________________________________________________________________________________________________________________________________________________________

34. On a scale of 1-5, how do you rate the security in your property? 1
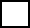
 2
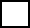
 3
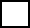
 4
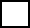
 5
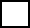


35. Are other anti-poaching personnel involved in bushmeat poaching?

Yes No

36. What form of assistance does those involved, give to bushmeat poachers? __________________________________________________________________________________________________________________________________________________________________________________________________________________________________________________________________________________________________________________

37. How best can Save Valley Conservancy address bushmeat poaching? ____________________________________________________________________________________________________________________________________________________________________________________________________________________________________________________________________________________________________________________________________________________

**NOTES:____________________________________________________________________________________________________________________________________________________________________________________________________________________________________________________________________________________________________________________________________________**
